# The effect of a community-based e-health program to promote the role of physical activity among healthy adults in Hungary

**DOI:** 10.1186/s12889-020-08750-1

**Published:** 2020-08-17

**Authors:** Alexandra Makai, Kata Füge, Zita Breitenbach, József Betlehem, Pongrác Ács, Kinga Lampek, Mária Figler

**Affiliations:** Faculty of Health Sciences, University of Pécs, Vörösmarty u. 4, Pécs, H-7621 Hungary

**Keywords:** Web-based, Physical activity, Health, Healthy adults

## Abstract

**Background:**

Physically active lifestyle can prolong the years spent without chronic diseases and is strongly associated with good mental and physical health. The goal of the study was to examine the physical activity patterns of the healthy adults and the effectiveness of a community-based e-health program.

**Methods:**

The study sample comprised of 633 participants. Analyses were based on the E-Harmony health program that was conducted in Hungary in 2014–2015. The longitudinal study measured the physical activity patterns of the healthy adult population, and a 12-month community-based internet and media program was evaluated for the target group to improve the physical activity level and related knowledge. We examined the effectiveness of the program by the International Physical Activity Questionnaire – Hungarian validated long version adjusting for socio-demographic parameters, also across multivariate linear regression analysis using SPSS 24.0 software. Confidence interval of 95% was used and the level of significance was *p* < 0.05.

**Results:**

The total physical activity of the study sample was 5129.9 (SD = 4488.1) MET min/week. Male participants scored higher in total activity but the results showed no statistical significance. Our participants were sitting 2211.6 (SD = 1592.8) min /week on average (daily average was 315.9 (SD = 227.6) minutes); the results showed no statistically significant difference by gender. We found weak but significant relationship between the active lifestyle and anthropometric data, especially according to leisure time activities and sedentary behaviour (*p* < 0.001). Based on the multivariate linear regression models the socio-demographic parameters significantly affected the physical activity level of participants. After the 12-month community-based e-health program, 10.34% changes occurred in the total physical activity level (*p* < 0.001).

**Conclusions:**

To our knowledge, ours was the first community-based e-health program in Hungary to improve the physical activity level of the healthy adult population. Based on our results this web-based e-health program can be an adequate tool to promote healthy lifestyle. The study could provide appropriate information for the further health interventions and policy making. Further research is necessary to determine the special risk groups and to develop an applicable e-health program for such specific subgroups.

## Background

Physically active lifestyle can prolong the years spent without chronic diseases and is strongly associated with good mental and physical health [1–7]. According to the World Health Organization’s (WHO) recommendation, at least 150 min of moderate physical activity per week is suggested for a healthy adult aged 18–64 [8]. According to the recommendation, activities aimed to improve muscle strength and endurance should be carried out 2–3 days a week [8–10].

The Eurobarometer 2017 data showed that 53% of the Hungarian population do no physical activity at all. 14% of Hungarians rarely engage in physical activity, and only 33% do sports regularly (either at professional or amateur level). While this result showed an improvement compared to the 2009 data, values are still lower than the European average [11]. According to the National Nutrition Survey 2014 (OTÁP), the proportion of overweight and obese adults in Hungary was 65% [12]. The WHO supports the evaluation of the community-based interventions to combat physical inactivity. Furhermore it defined e-health definitions also and communication strategies for health which can be used as an additional method to reach people [13, 14].

In addition, interventions and prevention programs aim to improve the health status of the population, with many methods available. Nowadays web-based prevention programs are becoming more and more popular with the widespread availability of the Internet and the frequent use of social networking websites. Studies confirmed the fact that internet-based prevention programs are easy to organize and flexible, with the great advantages of being available 24 h a day and their lower costs [15–19].

According to the data of the Hungarian Central Statistical Office, 66 and 78% of the population used the Internet regularly (at least once a week on average) in Hungary in 2011 and in 2016, respectively. Based on the above, the web- and social networking sites can be suitable for reaching the healthy adult population [20].

The healthy, working-age population (18–64 aged) is an important target group for health promotion [21] in the developed ageing European societies, as their health status and prolonged labour force is an important element for the economy [1, 22].

Self-care behaviors have an important function in the promotion of physically active lifestyle.

Our study is based on Pender’s Health Promotion Model and Bandura’s Social Cognitive Theory, both frequently used in measuring changes in physical activity patterns. Lifestyle and health behaviour is driven by personal conditions like socioeconomic or health status [23, 24].

The aim of our study was to explore Hungarian healthy adults’ current physical activity patterns and to measure the effectiveness of a community-based e-health program (E-Harmony Study) in increasing self-reported physical activity levels in individuals in a 12- month timeframe.

## Methods

### Study design and settings

Our study was an interventional pre-post design research using purposive sampling, representative for gender and age based on country statistics of the Hungarian Central Statistical Office. The target group was selected with the aim of examining the physical activity patterns and effectiveness of a community-based e-health program among healthy adults according to socioeconomic status. Baseline data collection occurred in June to September 2014 and the follow-up data derived from June to September 2015 in Baranya County, Hungary, among healthy 18–64-year-old adults.

A total sample of 750 participants was recruited and 633 individuals agreed to participate in the “E-Harmony” Study (response rate was 84.4%). Eligible participants reported good or very good health status and Baranya county residency, were not hampered in physical activities, and were aged 41.03 ± 13.25 years. Data were collected by trained researchers using standard tablet-assisted personal interviews, covering physical activity and socioeconomic parameters. Participating researchers were trained before the examination by a senior researcher team. All participants provided written informed consent prior to the survey. There was no incentive to participate in this examination.

### Measures

The first part of the data collection tool contained a demographic questionnaire with additional queries about health status.

The second part consisted of the International Physical Activity Questionnaire (IPAQ) – long version. Its Hunagrian version (IPAQ-HL) was validated among healthy adult population. The IPAQ long version contains 31 questions; the examined 5 domains were work, active transportation, household work, leisure time activitites, and sitting time. All activities were measured over the 7 days prior to data provision. We applied the IPAQ data truncation rule, and all activities exceeding 3 h were recoded as 180 min. Evaluation of physical activity has two different methods, using the frequency values as min/week scores or MET min/week values [25]. For summarizing physical activities, we calculated domain scores (MET min/week), and intensity scores as moderate, vigorous and MVPA values (MET min/week). The MET values are given in the study by Ainsworth et al. (2000) and the IPAQ scoring protocol. One MET is equal to energy expenditure during rest, and is approximately equal to 3.5 ml O2/kg min in healthy adults [26].

To measure antrophometric data we used the OMRON BF511 body composition monitor.

While measuring body weight (kg), participants were dressed in light clothing without shoes. Height (cm) was measured and recorded to the nearest 0.5 cm, in standing position, without shoes, using an anthropometric tape measure.

### Health and physical activity promotion program

E-Harmony is a community-based e-health program organized to encourage and guide active lifestyle. It is based on two popular and frequently used theoretical frameworks for health programs aimed at increasing physical activity. One is Bandura’s Social Cognitive Theory and the other is Pender’s Health Promotion Model [23, 24]. Health promotion is aimed at changing social, environmental, and economic conditions to improve the health of the target group or community (WHO, 2017). In 1973, Bandura’s experiments underpinned the theory of social learning, based on many interventions aimed at increasing physical activity today (Bandura, 1986). The theory claimed that most human behaviour is learned observationally through modelling, observing others and on later occasions this coded information serves as a guide of action. The theory explains human behaviour in terms of continuous reciprocal interaction between cognitive, behavioral, and environmental influences [27].

The Health Promotion Model was developed by Pender in 1996. It claims that health behaviour is driven by behaviour-specific conditions and affect interpersonal influences [24].

It focuses on helping people achieve higher levels of health-related quality of life and identifies background factors influencing that [28].

We evaluated our community-based e-health program based on Bandura and Pender’s theories. The first part of our research was a quantitative analysis of participants, where they completed a demographic and physical activity questionnaire. After the first data collection the research team summerized the results and evaluated the information dissemination and physical activity programs.

The program highlighted freely available outdoor, home, and work activitites and active transportation options. E-Harmony was developed by our research group and designed especially for the targeted population of healthy adults taking into account their socio-cultural specificities.

The program contained two elements as web-based video tools to increase physical activity level of the participants with 10 sessions and web-based educational contents to increase the physical literacy levels. Program elements were compiled by a research team consisting of an internal medicine specialist, physiotherapists, sport experts, and psychologists.

In the program videos we highlighted special interest groups like different age groups (middle aged and ageing adults), current sport trends for younger people and water activitites. Furthermore, we provided information on a training program applicable during healthy pregnancy and after childbirth.

In our health promotion program the physical activity videos were supplemented by training notes to increase the physical literacy of participants. Through our research website and social network sites our physiotherapist staff provided useful information on sports and physical activity topics. The purpose of the various educational contents was to draw attention to the importance of physical activity, especially on the tools and forms of exercise that are easily accessible and free of charge. Furthermore, every participant was mentored by a trained interviewer deriving from academic field of health sciences.

### Statistical analysis

Descriptive analyses were conducted to characterize the sample. The frequency numbers (%, N) were described the categorical variables and mean + standard deviation (SD) or mean + confidence interval (CI) the continuous variables. To examine results we used normality tests (Kolmogorov-Smirnov tests), non-parametric tests, Wilcoxon test (to measure changes in physical activity level), Mann-Whitney U tests (to measure group differences), and Sperman’s rank correlation (to measure the relationship between the different continuous physical activity data and antropometric and socioeconomic variables). To compare the categorical data, chi-square test was applied. The effect of socioeconomic parameters on physical activity was measured by multivariate linear regression analyses. Confidence interval of 95% was used and the level of significance was *p* < 0.05. Analyses were performed using SPSS 24.0.

## Results

### Socioeconomic and anthropometric characteristics of the study population

The major socioeconomic parameters of the study population are presented in Table 1. Half of the sample was married, two third of them had a job, and only 25% of the sample had a physically demanding job. Most of them had urban residence, and their educational level was secondary. We found significant differences in gender according to the marital status, work form, and financial situations (Table 1).

**Table 1:** General characteristics of the sample (*N* = 633)

		Total		Male		Female		χ2	p
		%	N	%	N	%	N		
Marital status	Single	**29.8**	186.0	**31.3**	97.0	**28.3**	89.0	16.5	**0.002**
	Married	**49.8**	311.0	**51.6**	160.0	**47.9**	151.0		
	Cohabitating	**11.5**	72.0	**12.6**	39.0	**10.5**	33.0		
	Divorced	**6.4**	40.0	**3.9**	12.0	**8.9**	28.0		
	Widow	**2.5**	16.0	**0.6**	2.0	**4.4**	14.0		
Working status	Yes	**70.8**	448.0	**77.3**	242.0	**64.4**	206.0	12.8	**< 0.001**
	No	**29.2**	185.0	**22.7**	71.0	**35.6**	114.0		
Type of the job	Sitting work with less than 30 min activitites	**26.3**	118.0	**24.4**	59.0	**28.6**	59.0	1.1	0.589
	Sitting work with more than 30 min activitites	**41.3**	185.0	**42.1**	102.0	**40.3**	83.0		
	Light work	**32.4**	145.0	**33.5**	81.0	**31.1**	64.0		
Age group	18–29	**23.7**	150.0	**24.3**	76.0	**23.1**	74.0	1.9	0.749
	30–39	**23.4**	148.0	**24.3**	76.0	**22.5**	72.0		
	40–49	**21.1**	134.0	**22.0**	69.0	**20.3**	65.0		
	50–59	**21.8**	138.0	**20.8**	65.0	**22.8**	73.0		
	60–64	**10.0**	63.0	**8.6**	27.0	**11.3**	36.0		
Place of residence	Rural	**10.4**	66.0	**10.5**	33.0	**10.3**	33.0	0.1	0.924
	Urban	**89.6**	567.0	**89.5**	280.0	**89.7**	287.0		
Education	High	**37.8**	238.0	**34.7**	108.0	**40.8**	130.0	2.4	0.119
	Middle	**62.2**	392.0	**65.3**	203.0	**59.2**	189.0		
Financial status	Lower than average	**49.0**	276.0	**46.1**	130.0	**52.0**	146.0	9.0	0.011
	Average	**25.6**	144.0	**23.0**	65.0	**28.1**	79.0		
	Higher than average	**25.4**	143.0	**30.9**	87.0	**19.9**	56.0		

Key element of our study was the examination of the antrophometric properties. The results are shown in Table 2. We found significant difference by gender according to all parameters except for waist circumference (Table 2).

**Table 2:** Antrophometric properties of the examined sample (*N* = 633)

	Total		Male		Female			
	Mean	SD	Mean	SD	Mean	SD	Mann-Whitney U	p
Weight	**78.2**	18.3	**86.3**	16.2	**70.2**	16.6	20,902.5	**< 0.001**
Height	**171.8**	9.1	**178.1**	6.9	**165.7**	6.5	9139.5	**< 0.001**
BMI	**26.4**	5.5	**27.1**	4.6	**25.7**	6.1	38,026.0	**< 0.001**
Waist circumference	**90.4**	15.5	**96.4**	14.2	**84.6**	14.5	25,827.5	**< 0.001**
Hip circumference	**103.0**	12.1	**103.4**	11.1	**102.7**	13.1	44,533.5	0.076
Fat %	**30.4**	9.9	**25.9**	8.8	**34.7**	9.0	22,501.5	**< 0.001**
Muscle %	**31.0**	6.1	**34.6**	5.4	**27.4**	4.4	13,637.5	**< 0.001**

### Descriptive analysis of physical activity patterns of the study sample

The physical activity patterns of the sample were measured by IPAQ-HL. After summarizing the scores, the main results were scored in continuous variables. These indicators were total physical activity, domain scores (as work, transportation, household activities, and leisure time actitivites), and intensity scores (moderate and vigorous physical activity and walking scores), all of them expressed in MET min/week. Respective results are presented in Table 3.

**Table 3:** Baseline physical activity patterns of the study sample (*N* = 633)

MET min/week	Male			Female			Mann-Whitney U	p
	Mean	CI		Mean	CI			
Work	**2136.3**	1734.3	2538.4	**1234.1**	913.5	1554.7	39,679.5	**< 0.001**
Transportation	**755.7**	643.9	867.5	**971.9**	849.4	1094.4	41,647.0	**< 0.001**
Household	**1317.6**	1105.7	1529.6	**1966.3**	1702.6	2230.0	39,331.5	**< 0.001**
Leisure time	**1104.4**	910.1	1298.7	**777.5**	652.4	902.6	47,682.5	0.282
Total	**5314.0**	4794.2	5833.9	**4949.8**	4477.1	5422.5	49,178.5	0.695
Moderate PA	**2565.3**	2248.5	2882.0	**2853.0**	2507.7	3198.3	46,704.5	0.142
Vigorous PA	**1856.4**	1526.0	2186.8	**976.4**	743.5	1209.3	40,074.0	**< 0.001**
Walking	**892.4**	784.6	1000.2	**1120.5**	1008.3	1232.7	42,737.5	**0.001**
Total weekly sitting time (min)	**2280.1**	2112.9	2447.2	**2144.6**	1960.3	2329.0	46,845.0	0.160
Daily sitting time (min)	**325.7**	301.8	349.6	**306.4**	280.1	332.7	46,845.0	0.160

The total activity was 5129.9 (SD = 4488.1) MET min/week. Male participants scored higher in total activity but the results did not show statistical significance. The total scores contained all examined domains of the IPAQ-HL as listed before. Two third of the activities were work and household activities and only one third of them were leisure time activitites and active transportation (i.e. walking and cycling).

Another indicator in examining the active lifestyle is sedentary behaviour and time spent sitting. On average, participants were sitting 2211.6 (SD = 1592.8) min/week. The daily average time spent sitting was 315.9 (SD = 227.6) minutes. The results did not show statistically significant difference among genders.

Physical activity patterns were described for the whole sample and for male and female participants separately. Male adults were more active in work and in leisure time activitites, while female adults spent more time on household activitites and active transportation. According to the intensity of the activitites, male participant spent more time with vigorous activitites than females (Table 3).

### Comparative analysis of physical activity and anthropometric parameters

The results in Table 4 showed correlations between antrophometric properties and physical activity patterns. We found weak but significant relationship between the active lifestyle and antrophometric data, especially according to the leisure time activities and sedentary behaviour. The physically active lifestyle has a positive effect on body composition scales (Table 4).

**Table 4:** Spearman’s Rank Correlation between baseline IPAQ-HL measures and anthropometric data

MET min/week		BMI	Waist circumference	Hip circumference	Fat %	Muscle %
Work	R	0.069	0.087	0.007	−0.127	0.172
	p	0.085	**0.031**	0.862	0.001	0.000
Transportation	R	− 0.137	− 0.128	− 0.070	0.011	− 0.049
	p	**0.001**	**0.001**	0.081	0.775	0.222
Household	R	0.073	0.042	0.071	0.148	−0.152
	p	0.069	0.292	0.078	**< 0.001**	**< 0.001**
Leisure time	R	−0.151	− 0.175	− 0.137	− 0.188	0.188
	p	**< 0.001**	**< 0.001**	**0.001**	**< 0.001**	**< 0.001**
Total	R	−0.020	−0.021	− 0.030	− 0.078	0.084
	p	0.616	0.600	0.461	0.053	**0.035**
Moderate PA	R	0.053	0.025	0.015	0.029	−0.015
	p	0.184	0.538	0.716	0.477	0.710
Vigorous PA	R	−0.056	− 0.036	−0.092	− 0.223	0.247
	p	0.164	0.374	**0.022**	**< 0.001**	**< 0.001**
Walking	R	−0.112	−0.130	− 0.063	− 0.002	−0.037
	p	**0.005**	**0.001**	0.117	0.958	0.355
Total weekly sitting time (min)	R	0.096	0.110	0.087	0.099	−0.079
	p	**0.016**	**0.006**	**0.029**	**0.013**	**0.049**
Daily sitting time (min)	R	0.096	0.110	0.087	0.099	−0.079
	p	**0.016**	**0.006**	**0.029**	**0.013**	**0.049**

### Multivariate analysis of physical activity level and the predicting socioeconomic parameters

In our study we examined the effect of the sociodemographic parameters on physical activity (PA) patterns with multivariate linear regression analysis. We analysed 5 regression models where the dependent variables were total PA, work PA, active transportation, household activitites, and leisure time activitites. We found significant relationship between the physical activity pattens and sociodemographic variables such as age, education, work type and financial status, results of which are shown in Table 5.

**Table 5:** Multivariate regression analyses on social determinants of physical activity patterns

		Work	Transportation	Household	Leisure time	Total
R^2^		0.3	0.0	0.1	0.0	0.2
F		95.9**	5.9*	12.2**	5.7*	38.8**
B constant		5007.3**	715.9**	1714.8**	972.3**	8077.5**
B	18–29 aged			- 975.1**		- 1211.4*
	40–49 aged		253.7*		- 361.4*	
	Female			485.3*		
	Education - high	1827.1**		760.0**		2479.8**
	Light work				- 313.3*	
	Sitting work	- 3369.0**				- 3019.9**
	No financial problems		- 209.3*			
	Average financial status				- 316.8*	

*p < 0.05, **p < 0.001

### Effectiveness of the community-based e-health program by physical activity level

We found significant positive effect of active lifestyle on anthropometric properties and our results showed that socio-demographic parameters significantly affected the physical activity level of Hungarian healthy adults.

We examined changes after the 12-month web-based health promotion program. 10.34% changes were shown in total physical activity level. The different subdomains showed the details of changes in PA patterns: the vigorous activity values did not increase (−4.5%), while the highest increase was found in walking (36.5%), transportation (27.7%), and leisure time activities (16.1%). The increased level of PA and the decreased level of sitting time (23.4% weekly sitting time in total) was evaluated (Table 6).

**Table 6:** The examination of the effect of a 12-month community-based e-health program among healthy Hungarian adults (*N* = 497)

	Baseline mean	Baseline SD	Follow up mean	Follow up SD	Change %	Z	p
Work	1866.0	3485.0	2282.6	3423.4	18.3%	−14.219	**< 0.001**
Transportation	862.2	1050.7	1192.1	1369.7	27.7%	−17.595	**< 0.001**
Household	1613.1	2083.5	1872.2	2265.3	13.8%	−17.993	**< 0.001**
Leisure time	954.8	1471.1	1137.8	1583.3	16.1%	−15.538	**< 0.001**
Moderate PA	2750.2	2952.4	2822.8	2903.2	2.6%	−18.585	**< 0.001**
Vigorous PA	1500.5	2695.9	1436.4	2358.4	−4.5%	−13.523	**< 0.001**
Walking	1045.5	1018.3	1647.7	1560.8	36.5%	−18.566	**< 0.001**
Total PA	5296.2	4554.0	5906.9	4791.2	10.3%	−19.529	**< 0.001**
Total weekly sitting time (min)	2591.5	2066.0	2099.9	1351.1	−23.4%	−19.543	**< 0.001**

## Discussion

In our study we measured the effect of an online community-based e-health program.

The 12-month community-based e-health program could be considered successful, as 10.34% changes were shown in total physical activity level (*p* < 0.001). Our findings support further development of additional specific interventions for different target groups.

Based on our results the community-based e-health program could be a popular and effective way to motivate healthy adults to follow a healthy and physically active lifestyle.

In the past years several studies were published to examine the effectiveness of different PA health programs and interventions [29]. Most of the research emphasized that web-based interventions are less effective than personal communication, but their important advantage is extensive availability [30]. Furthermore, web-based interventions are popular among the participants also, because more than half of the health Internet users would like to increase physical activity across online health programs [31].

The role of personal coaching is beneficial in motivation but the educational role is indispensable in health programs, when it comes to training participants on precise execution. This fact was proven in our study and several other ones in past years.

Nakamura et al. sought to increase the popularity of vegetable consumption among adult population. Their research emphasizes that Internet-based surveys nowadays have the advantage of reaching lower socio-demographic groups [32].

The active adulthood of the population can be achieved by maintaining the physical activity in age-appropriate form. In their research Braun et al. drew the attention of the target group on educational materials (videos and instructional films) during the intervention. As a result, participants could retain their physical independence longer and can continue to participate in the labour market [33].

Larsen and coworkers created a web-based physical activity intervention program for adolescents with participants who exercised less than 90 min per week. They participated in a 12-week program with online intervention. Questionnaires and accelerometers were applied to analyze the efficacy of the intervention. All in all, Larsen’s research has proven that smart phone applications and Internet-based research are appropriate and effective [34].

Larsen et al. also developed a web-based physical activity enhancement program for adults, with 205 adults aged 18–65, in the United States. The aim of the program was to increase physical activity by providing participants with online tutorials. Through the website, it was possible to set goals, measure activity and communicate with participants, according to the proven results of the program [35].

Degroote et al. developed a research that introduced MyPlan 1.0, to initiate behavioral change and increased physical activity with 328 participants. The program brought about significant changes in overall moderate and vigorous physical activity. However, as a disadvantage of the program, the authors indicated a high dropout rate, more than half of the respondents dropping out during the study [36]. In our study the dropout rate during 12 months was 21.48%, which could be considered a successful result, and is probably due to the personal coaching element of the program.

In our study we evaluated physical activity levels by IPAQ-HL questionnaire using tablets by trained interviewers. We found that physical activity patterns are strongly correlated with sociodemographic and anthropometric parameters.

According to our results, the physical activity level of the healthy Hungarian adult population is strongly determined by work and household activitites, while one-fifth of the total scores were leisure time activities including walking, moderate, and vigorous activities. European data, expecially the V4 data showed similar results (total PA score of V4 countries was: 6023.95 ± 5727.01), but the Hungarian scores showed lower values, thus a suitable aim could be to catch up with the European average [11, 37, 38].

Our results confirmed that the more time spent with sitting, the less favourable the anthropometric data was, and the more time spent with leisure sports, the better the anthropometric data was, which clearly shows the role and significance of physical activity in body composition data.

To define the sociodemographic predictor factors of the different physical activity domains of the study population, we calculated 5 multivariate linear regression models where total physical activity and IPAQ-HL domains (work, transportation, household and leisure time activity scores) were the dependent variables and sociodemographic parameters were the predictor variables.

The research confirmed that individuals’ physical activity decreases with the increasing of age. Elderly people spend less time with movement, which in some cases might be related to the lack of information on active transport options [39–42]. Besides, several studies have shown that young adults are less active than middle-aged people [43, 44].

The participants with terciary education were more active than others, which was confirmed by other studies that examined the effect of education on physical activity level. Secondary or lower education means more activitites in work time because of the type of work but the amount of physical activity in leisure time activitites were proven to be higher in case of persons with high education [21, 43, 45–51]. Education is tightly bound to financial status and work type. Sedentary work, unemployment, and retirement are all showing lower activity patterns [52, 53].

The physical activity studies presented different results by gender. Most of the studies reported more active lifestyle of men but the result of our regression model showed that female participants are more active in household activitites [43, 45–49, 54].

Compared the 2014 and the 2015 results, our study showed that although there were no major changes in case of sports, participants’ efforts showed that they spent less time sitting, increased activity in front of their desk, and they also walked more during work time. A more spectacular change was the increasing popularity of walking among modes of transport.

An important element for our target group was to emphasize the link between sustainable, active transport and physical activity and health, and to increase the health and physical literacy among all socioeconomic groups [55, 56].

However, when examining the relationship between socio-demographic factors and physical activity, activity levels depend on certain demographic characteristics, confirming the need to take this information into account when designing physically active lifestyle programs and to pay greater attention to specific target groups.

## Limitations

The limitations of the study include the fact that the sample was not randomized and there was no control group. The measurement of the physical activity level was examined by a subjective quantitative method by IPAQ-HL questionnaire. The objectively measured physical activity level could present precise information but in our study there was no financial or technical possibility to adapt this method.

## Conclusion

The online community-based e-health program could provide an appropriate way to promote health and physically active lifestyle among healthy adults in Hungary. Based on the results of our study, physical activity level strongly correlated with socioeconomic and anthropometric parameters, which should be considered in case of the development of a health and physical activity promotion program.

## Data Availability

The dataset supporting the conclusions of this article is available from the corresponding author on reasonable request.

## Abbreviations

BMI: Body mass index
CI: Confidence interval
IPAQ-HL: International physical activity questionnaire (IPAQ) – Hungarian long
KSH: Hungarian central statistical office
MET: Metabolic equivalent of task
OTÁP: National nutrition survey
PA: Physical activity
SD: Standard deviation
WHO: World health organization
